# Advancing evidence‐based practice through the Knowledge Translation Challenge: Nurses’ important roles in research, implementation science and practice change

**DOI:** 10.1111/jan.16362

**Published:** 2024-08-01

**Authors:** Amanda Chisholm, Angela Russolillo, Michelle Carter, Marla Steinberg, Leah Lambert, Andrea Knox, Agnes Black

**Affiliations:** ^1^ Vancouver Coastal Health Research Institute Vancouver British Columbia Canada; ^2^ Providence Health Care Vancouver British Columbia Canada; ^3^ Evaluation & KT Consultant and Educator Victoria British Columbia Canada; ^4^ BC Cancer Vancouver British Columbia Canada

**Keywords:** capacity building, evidence‐based practice, health care professionals, implementation science, knowledge translation, nursing

## Abstract

**Aim:**

To describe a knowledge translation capacity‐building initiative and illustrate the roles of nurses in practice change using an exemplar case study.

**Design:**

The report uses observational methods and reflection.

**Methods:**

The Knowledge Translation Challenge program involves a multi‐component intervention across several sites. The advisory committee invited eligible teams to attend capacity‐building workshops. Implementation plans were developed, and successful teams receive funding for a 2 year period. Evaluation involved collecting data on program uptake and impact on practice change. Data has been collected from five cohorts. The exemplar case study employed an action‐research framework.

**Results:**

Four nurse‐led teams have demonstrated successful implementation of their practice change. The case study on implementing a clinical toolkit for clozapine management further illustrates a thoughtful planning process, and implementation journey and learnings by a team of nurses.

**Conclusion:**

The Knowledge Translation Challenge program empowers nurses to use implementation science practices to enhance the quality and effectiveness of healthcare services. Success of this initiative serves as a model for addressing the persistent gap between knowledge and practice in clinical settings and the value of activating nurses to help close this gap.

**Implications:**

As the most trusted and numerous profession, it is vital that nurses contribute to efforts to translate research evidence into clinical practice. The Knowledge Translation Challenge program supports nurses to lead practice change.

**Impact:**

The Knowledge Translation Challenge program successfully equips nurses and other health care providers with the knowledge, skills and resources to implement practice improvements which enhance the quality and effectiveness of healthcare services and nursing practice.

**Patient or Public Contribution:**

The Knowledge Translation Challenge advisory committee has three patient‐public partners that support teams to develop a patient‐oriented approach for their projects by providing feedback on the implementation plans. Each team was also supported to include patient‐public partners on their project.

**What does this paper contribute to the wider global clinical community?**

- Highlights the KT Challenge program as a successful initiative in advancing Implementation Science within nursing.
- Suggests a scalable approach for other healthcare organizations aiming to enhance their nursing workforce's capacity for evidence‐informed care.
- Results contribute to the broader academic discourse on implementing evidence‐based practices in complex healthcare settings.

## 1 INTRODUCTION

Nurses are uniquely positioned within the healthcare system to identify and implement evidence‐based practice changes. As the largest and consistently most trusted members of the healthcare workforce (NASEM, 2021), nurses play an important role in reducing the persistent gap between what we know (knowledge) and what we do (clinical practice). As a profession, nurses engage extensively with patients, increasing opportunities to identify existing gaps and champion the integration of evidence‐based clinical practices benefiting patients and the larger health care system.

Implementation Science (IS) is defined as ‘the scientific study of methods to promote the systematic uptake of research findings and other evidence‐based practices into routine practice, and hence, to improve the quality and effectiveness of health services and care’ (Eccles & Mittman, 2006, p. 1). The IS field is growing and it is crucial for nurses to contribute to its advancement and use evidence‐based implementation science practices to support the uptake of evidence‐based practices. Both nationally and internationally the role of nurses is expanding with increasing recognition of their importance and unique contributions to the field of IS. Nurses are progressively taking on roles as clinical champions or change agents within healthcare organizations (Santos et al., 2022, White, 2011), and organizations are developing nurse‐led IS specialist programs to spearhead the implementation of evidence‐based practices and interventions at the bedside (Russell‐Babin et al., 2023). Moreover, advanced practice nurses, including nurse educators, nurse practitioners and clinical nurse specialists, are well positioned to identify, implement, and evaluate practice change efforts (Zullig et al., 2020), thus contributing to the field of IS. Involving direct‐care nurses in practice change using the implementation science paradigm can improve their skills to engage in evidence‐informed practice, increase job satisfaction, quality of care, patient outcomes and organizational service efficiencies (Black et al., 2015; Orrell et al., 2015; Rana et al., 2022; Ruco et al., 2021).

Scholars have emphasized the potential for nursing to enhance its impact in healthcare by “embracing implementation science as a central research paradigm” (Zullig et al., 2020, p. 4), arguing that by using IS, valuable nursing‐led improvements can be optimized (Roberts et al., 2023). Despite the critical value of nurses in this capacity, there are limited opportunities for nurses to gain IS expertise. A recent meta‐synthesis called on health care organizations to adequately fund and make continuing professional development accessible to nurses as a critical step toward promoting professionalism, continued education, and improving patient care standards (Mlambo et al., 2021). IS training takes a dedicated effort from health care organizations and it should be considered as a key component of professional development opportunities for nurses.

## 2 BACKGROUND

In order to address the gap in professional development opportunities for IS within nursing and other health care professions, two health care organizations developed the Knowledge Translation (KT) Challenge program, based on a research capacity building program for clinicians. Since 2017, the KT Challenge has supported nurses and other health care professionals to effectively implement evidence‐based practices. The KT Challenge has been found to be an effective intervention for increasing the capacity (e.g., knowledge, skills and confidence) of health care providers to implement evidence‐based practice changes (Black et al., 2021). In 2022, the KT Challenge program expanded to a third health care organization to support the professional development of its nursing workforce. These organizations recognize that creating a strong foundation for development and engagement in IS will support rapid integration of new knowledge that will improve clinical practice, promote evidence‐informed care, and enhance nursing practice.

The multi‐health care organization partnership model has benefits to program delivery, including expanding the pool of mentors and patient‐public partners and enhanced learning for all teams through exposure to projects and practice areas outside their established network of health care professionals. This provides nurses with a deeper and more collaborative learning experience that helps broaden their perspectives on how knowledge translation takes shape within the healthcare setting.

## 3 THE STUDY

### 3.1 Study aims

This study aimed to describe a knowledge translation capacity‐building initiative and illustrate the roles of nurses in leading an evidence‐based practice change using an exemplar case study.

## 4 METHODS

### 4.1 Study design and setting

The KT Challenge is an annual program conducted across three health care organizations. The case study described below highlights a nurse‐led implementation project undertaken as part of the KT Challenge program in an acute mental health unit.

The KT Challenge program is supported by a leader and coordinator at each organization, with a dedicated workload equivalent to one day per week. The program organizers support promotion of the program at their sites, consult with teams, coordinate the workshops, manage the peer review process, monitor team progress, support teams to successfully navigate barriers, and lead the program evaluation. The organizations share the cost of a KT expert to design and deliver the workshops and review sections of the implementation plans.

### 4.2 Study intervention

**FIGURE 1:**
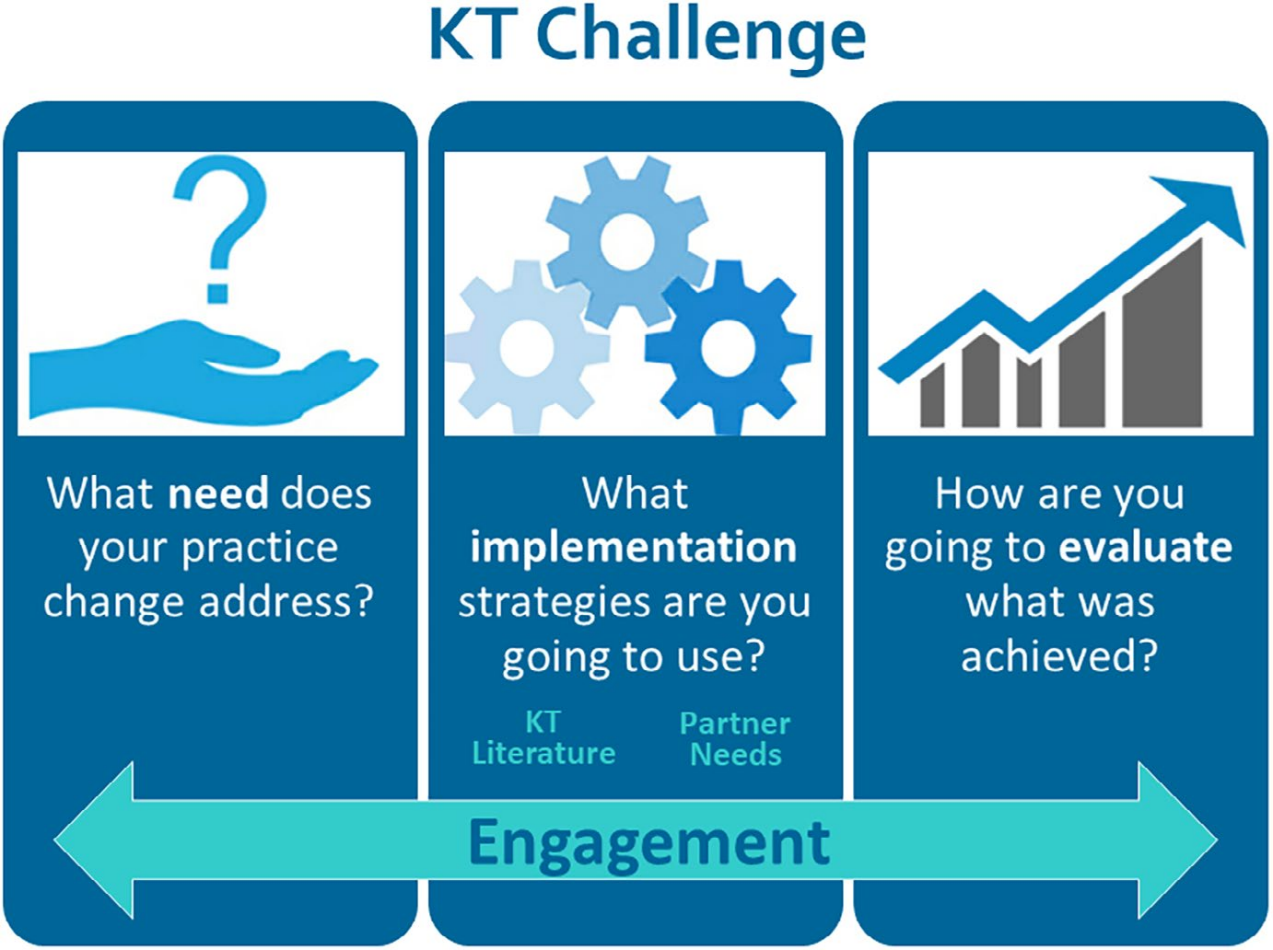
The KT Challenge application incorporates three pillars of implementation planning; demonstrate the need for the practice change, select the implementation strategies to support the practice change, and develop the evaluation plan to determine whether your practice change was successful in achieving your improvement.

The KT Challenge is a multi‐component implementation capacity building and support program that involves training, mentorship, peer review and funding. The process begins with a letter of intent (LOI) where teams of health care professionals describe the evidence‐based practice change they want to implement, document the need for this practice change, and summarise evidence of its effectiveness. The LOIs are reviewed by an advisory committee composed of program leaders, patient‐public partners, point‐of‐care practitioners and KT experts. After approval from the advisory committee, eligible teams are invited to attend a series of capacity building workshops, led by a KT expert, and grounded in IS theories, models, frameworks and research. The workshops provide learners with the knowledge and skills required to develop an implementation plan and evaluate the uptake of the evidence‐based practices (Figure 1). The workshops are centred around the implementation planning steps of engaging partners, assessing barriers and facilitators, selecting implementation strategies and evaluating the practice change. Workshop participants learn about key IS models, theories and frameworks including the International Association for Public Participation (IAP^2^, 2018) Spectrum of Public Participation, Consolidated Framework for Implementation Research (CFIR) (Damschroder et al., 2002), and the COM‐B model (Michie et al., 2011). Learners use the IAP^2^ continuum to identify appropriate roles for engaging partners; they review the CFIR to identify potential barriers and facilitators in advance of connecting with partners about barriers and facilitators, and they use the COM‐B model to design their implementation strategies to cover the main facets that support the desired behavioural change. The COM‐B model is also used to develop the evaluation plans ensuring that the evaluation includes indicators related to capability, opportunity, motivation and of course, behaviour. The workshops were delivered in‐person for the first four cohorts, then switched to a virtual platform. Project teams are required to collaborate with a KT mentor and patient‐public partner within their clinical area to support implementation and evaluation of the practice change. Project teams used feedback received on the workshop materials (e.g., engagement plans, barriers and facilitators worksheets and implementations strategies) to develop a full implementation plan for the final application and submitted it to the advisory committee for review. Then, approved teams were provided with $5000 in funding over a 2 year term to implement their evidence‐based practice change.

### 4.3 Data analysis

Data on team members (e.g., job title and practice area) in the KT Challenge program is collected at the LOI and final application stages. Team members were allowed to be added or changed between the LOI and final application. Teams were required to complete final reports on the impact of their evidence‐based practice change, including a description of how the practice change was implemented and evaluated. The case study described below illustrates a project led by a team of nurses to demonstrate uptake of their practice change.

## 5 RESULTS

### 5.1 KT challenge program evaluation

#### 5.1.1 Program uptake by nurses and health care providers

**FIGURE 2:**
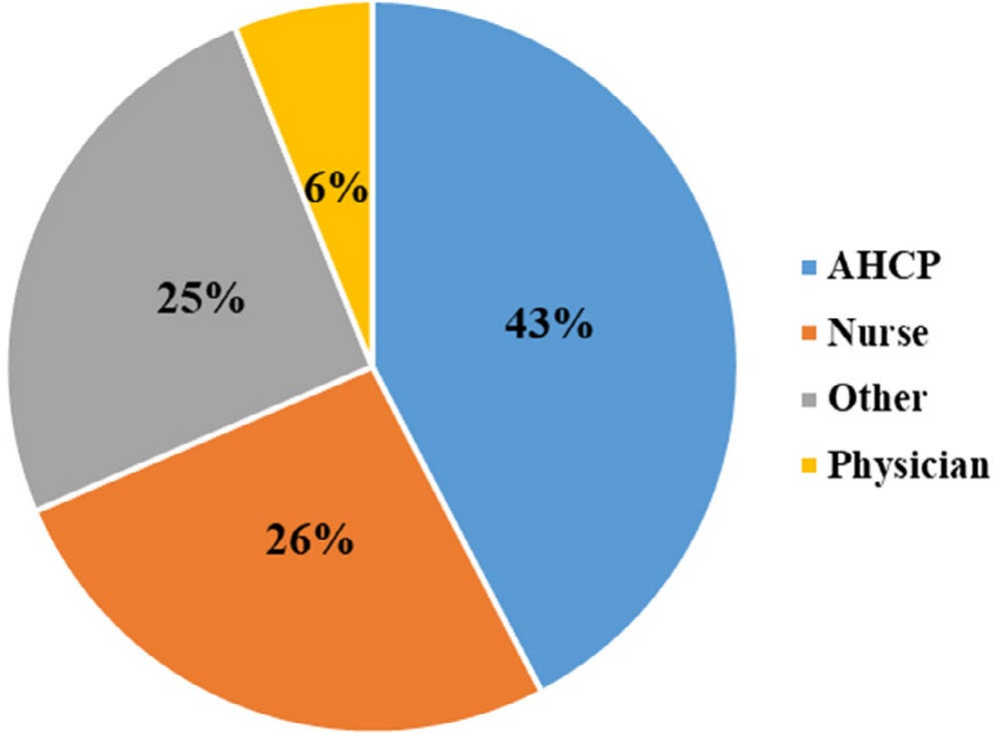
Participants on funded teams by health profession, including allied health care providers (AHCP), nurses, physicians, and others (*n* = 229).

The KT Challenge program began in 2017 and has recently reviewed applications and awarded funding to the seventh cohort. To date, 96 teams (31 with a nurse team leader) have submitted a LOI, and 52 teams (14 with a nurse team leader) have been funded. Nurses participated as KT mentors on 16/52 (31%) of the funded teams. Overall, nurses had a role as leader, mentor or core team member on 29/52 (56%) of the funded teams. Of the 229 participants across the funded teams, 43% were allied health care professionals, 26% nurses, 25% other health care staff (e.g., researchers, managers, coordinators, quality leaders and health systems advisors), and 6% physicians (Figure 2).

#### 5.1.2 Evidence‐based practice changes

Fifteen out of 30 teams across five cohorts were able to demonstrate the uptake of the intended practice change. Five additional teams collected anecdotal information on the uptake of the practice changes. Taken together, this results in a success rate of 66% which exceeds the rate of success reported across other KT capacity building initiatives (Durlak & Dupre, 2008). Seven teams are still actively conducting their projects, and three teams withdrew due to management changes and shifting priorities within the department. The remaining teams from the 2022–2023 cohort are still within their 2 year project term, and do not yet have data on uptake of practice change.

Teams with a nurse leader or mentor successfully led the implementation of a:

- clozapine clinical toolkit (CTK) in a psychiatry inpatient program (see case study);
- depression screening tool in a cardiac inpatient program;
- sepsis awareness initiative for early recognition and reduced admission from the emergency department and;
- substance use education to improve harm reduction practices in a psychiatry inpatient program.

### 5.2 Case study

#### 5.2.1 Description of project

An interdisciplinary team of nurses and pharmacists developed, implemented, and evaluated a CTK to promote the safe initiation, monitoring, and side effect management of patients prescribed clozapine, an atypical antipsychotic medication (Gessner et al., 2024). Current international guidelines endorse clozapine as the gold standard for treatment‐resistant schizophrenia (National Institute for Care Excellence, 2014; Pringsheim & Addington, 2017); however, structured monitoring is required due to risk of hematologic and cardiovascular side effects (De Berardis et al., 2012; Ronaldson et al., 2015). Despite clear practice recommendations, several studies have reported shortfalls in the delivery of clozapine care (Bolton, 2011; Mitchell et al., 2012).

Informed by action research methodology (Field et al., 2014, Graham et al., 2006) and IS, the development of the CTK involved a comprehensive current state assessment. The team began with a literature review to identify clozapine best practices, focusing particularly on prescribing and monitoring. A retrospective chart audit was conducted to understand site‐specific clozapine care practices.

Action research facilitated an iterative, collaborative process wherein key partners, including clinicians, patients, and administrators, actively participated in identifying challenges and co‐developing solutions. This participatory approach ensured that the CTK was evidence‐based and contextually relevant and aligned with the real‐world needs of its users. The team engaged in clinical consultations with key practitioners and patient partners to obtain input on the information gathered and to further define barriers and facilitators, challenges, needs and resources related to clozapine care. IS provided the framework for systematically integrating the CTK into clinical practice. This involved identifying potential barriers, outlining implementation plans, and creating strategies to sustain long‐term use of the toolkit.

Through the principles of IS (Bauer et al., 2015; Casey et al., 2018), the team ensured that the CTK was adaptable to various clinical settings, supported by continuous education and training for staff. Rigorous evaluation measures were established to assess the effectiveness of the CTK, enabling continuous refinement and improvement based on feedback and results. Together, action research and IS created a robust foundation for the CTK project, enabling thorough assessment, partner engagement, practical integration and sustained impact on clinical practice.

**FIGURE 3:**
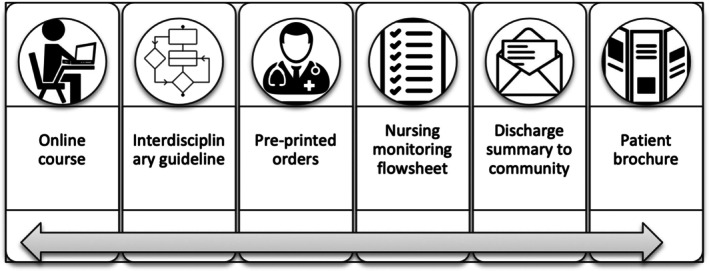
Clozapine clinical tool kit components.

The CTK aimed to promote safe initiation, monitoring, and management of patients prescribed clozapine. Portions of the CTK (e.g., guideline, pre‐printed orders, monitoring flowsheet) were adapted from a local affiliated hospital to meet the needs of the implementation site. Other elements (e.g., online course, medication discharge summary and patient handout) were created to augment existing tools. In total, the CTK consisted of six core components (Figure 3).

#### 5.2.2 CTK implementation

The CTK implementation team (*n* = 9) consisted of clinicians, researchers, and a project manager, with expertise in psychiatry, pharmacology, nursing, education and change management. The implementation process used multiple strategies, including extensive engagement, staff education and communication. As a prototype, the CTK was presented to clinicians across disciplines to validate content, design, and function. Revisions were made based on feedback related and re‐presented for finalization. In parallel to prototype validation and approval, the implementation team met with staff nurses, psychiatrists and pharmacists to seek input on proposed implementation activities such as CTK integration and staff education.

To promote seamless integration into practice, the team embedded toolkit components into existing clinical workflows and worked with the hospital's pharmacy dispensary to ensure clozapine order processing and dispensing required a completed pre‐printed order (PPO) set. Staff education consisted of a CTK online course, discipline‐specific in‐services, and in‐person practice support. The team also met with clinical leaders to discuss project updates and address potential barriers to CTK uptake (e.g., staff resistance, competing clinical priorities, etc.). Project team members scheduled daily visits to the inpatient units to provide in‐person practice support for the first two weeks of CTK use. They also audited charts to assess and address documentation challenges in real‐time.

#### 5.2.3 CTK results

A chart review was completed before and after CTK implementation. A total of 298 patient admissions between December 2016–2018 (185 pre‐CTK) and January 2019–2020 (113 post‐CTK) were analysed.

Prior to implementation (pre‐CTK) care did not meet best practice clozapine guidelines across several parameters including documentation of patient status (62%), medication adherence (50%), and baseline blood work and diagnostics (e.g., complete blood count (CBC) with differential, electrocardiogram (ECG), fasting blood glucose or A1c, lipid panel (50%); C‐reactive protein (CRP) and troponin (< 25%)).

In the year following implementation (post‐CTK), the clozapine PPO was utilised for 96% of patients prescribed clozapine, indicating a high level of clinical adoption. Baseline orders for all parameters (CBC, CRP, troponin, glucose, lipids and ECG) were above 50%. Moreover, there was a significant increase in the proportion of admissions with clozapine monitoring adhering to best practice more than 75% of the time (troponin 73.5%, CRP 73% and CBC with diff 75%).

#### 5.2.4 Reflections

This case study described the development, implementation, and evaluation of a clozapine CTK using action research, a framework used to facilitate change and generate new knowledge by involving researchers and practitioners (Leitch & Day, 2000; Lingard et al., 2008) and IS. The collaborative nature of the project was useful in bridging the evidence to practice gaps commonly described in IS (Westerlund et al., 2019). The CTK study benefited significantly from the multi‐organisation partnership model, which facilitated increased opportunities for cross‐site collaborations and the integration of standardised best‐practice evidence for clinical staff and patients. The interdisciplinary team, which included representatives from two of the three sites, ensured seamless implementation of the toolkit. Without the partnership several barriers would have impeded the teams' ability to bridge the critical gap between evidence and practice, thereby limiting the provision of necessary education and tools to clinicians for improved patient care. Throughout the project, team members expressed value in incorporating empirical findings, participant perspectives and clinical experience.

Prior to implementation, as per the implementation planning process taught in the workshops, our team reflected on ways in which unit culture and clinical workflows might impede or facilitate knowledge uptake. In consulting with key personnel, we identified competing priorities, limited resources and change fatigue as potential barriers. By embedding components of the CTK into existing workflows (automation), creating provisions to address CTK non‐compliance (safeguards), and highlighting potential outcomes of CTK use (impact), our team was able to address perceived barriers and integrate the CTK into a complex and dynamic inpatient setting. Leadership support for the CTK and the creation of suitable work conditions (e.g., time and space for staff to participate in development and education) were also important for implementation. We reinforced our integration efforts with three approaches to staff education—an online course, discipline‐specific in‐services and in‐person practice support—each conferring advantages and disadvantages. Discipline‐specific in‐services were well received but challenging to schedule.

Despite being resource‐intensive, the project resulted in a CTK reflective of best practices, tailored to our setting, and acceptable to clinicians. Substantial human resources were needed to develop, implement, and evaluate the project, including the necessary staff engagement, education and clinical support. Although the project was conducted on a relatively small budget with minimal direct costs, the required human resources were extensive. The collaborative nature of action research and IS facilitated overcoming setbacks and coping with uncertainties throughout the project. Lessons learned included the critical importance of clinician engagement, risk mitigation, sustaining adherence through education and acknowledging the significant time and personnel investment required for successful implementation. These lessons will inform future projects in the healthcare domain.

(1) *Clinician engagement*: Acknowledging the essential role of clinicians at every stage of this process was paramount to successful implementation. We actively engaged with nurses, physicians, and other healthcare professionals, recognizing the opportunity to embrace frontline expertise and practical insights throughout the project. By actively inviting user input, we created a collaborative atmosphere where professionals felt valued and fostered a sense of ownership among clinicians, instilling in them a commitment to successful CTK integration into practice.

(2) *Mitigating risks*: While a knowledge translation project may appear fairly low risk, commitment to risk evaluation and mitigation was critical. In the early phases of implementation planning, we reviewed and assessed potential challenges to implementation. Specifically, we conducted multiple risk assessments to anticipate and identify barriers to clinician engagement, CTK integration, project evaluation, and developed proactive strategies to mitigate these risks. This proactive approach not only supported the implementation of the CTK but also minimized disruptions in patient care.

(3) *Nurturing knowledge*: Investing in ongoing education was critical, especially in the context of an unpredictable workforce with respect to staff turnover. While the initial implementation phase focused on educating nurses, we proactively planned for continuous support and education to ensure ongoing staff capacity related to clozapine care and documentation. Planning to offer periodic “refreshers” served as a strategic investment, reinforcing foundational knowledge and best practices, while also providing an avenue in which to provide CTK updates as needed. Equally vital was the development of a comprehensive education plan tailored for new staff.

(4) *The gift of time*: The implementation and evaluation of the CTK demanded more time and use of human resources than originally anticipated. We recognised that rushing through any stage of implementation could result in oversights, potentially undermining the CTK's effectiveness and, ultimately, the quality of patient care. Consequently, we prioritized and adjusted the time allocated to all project phases from planning through to evaluation. Recognising the significant time investment required for CTK implementation was crucial and underscored the importance of patience and comprehensive planning to navigate the pathway toward successful practice change.

## 6 DISCUSSION

### 6.1 Uptake of the KT Challenge

The KT Challenge program has been shown to be successful at improving the capacity of health care professionals to lead evidence‐based practice changes by increasing their knowledge, skills and confidence after attending workshops that support the development of their implementation plans and evaluation plans (Black et al., 2021). In addition, the funded teams have been able to demonstrate the intended practice improvements. The program continues to see a steady number of new applications, including nurse‐led teams, and has expanded to a new health care organisation, indicating a growing interest among health care providers to mobilise knowledge into practice. Participants in the program have benefited from the expanded partnership opportunity by making connections with new mentors and patient‐public partners, learning from other practice areas and expanding their networks. Evaluation of the program demonstrates that IS training for nurses and health care providers can help close the evidence‐to‐practice gap by empowering those responsible to use new knowledge in practice.

Nurses represented a quarter of all participants in the KT Challenge program, and they frequently took on leadership roles, serving as team leaders and mentors. Nurses, as team members, leaders and mentors, were critical to the success of each project. Their integration on the clinical units and understanding of how knowledge should be shared and integrated into practice was essential to ensuring uptake of the practice changes. Moreover, the KT Challenge program provided the foundational support for nurses to lead and implement evidence‐based practice changes. It is important to empower nurses to deliver the best care possible, and providing training on the knowledge and skills to effectively move evidence into practice enables them to contribute meaningfully to implementation science research.

Our findings highlight several successful practice changes that resulted from participation in the KT Challenge program. Half of the teams reported demonstrable improvements in practice across a variety of settings (e.g., acute care, emergency care, outpatient services, community health centre), and another 16% of teams implemented their practice change but did not collect data on the uptake. Teams who were unable to complete their projects mentioned that participation in the KT Challenge program was still beneficial to gain knowledge and skills in IS.

### 6.2 Limitations of the program

Similar to other practice improvement programs in health care settings, a few teams faced challenges such as turnover among project team members and managers, lost communication with their mentor and delays in project completion. In response to the COVID‐19 pandemic, two teams withdrew from the program due to shifting priorities and limited capacity to add duties beyond direct patient care. The KT Challenge program leaders continue to seek feedback from participants on their experience and learn from the challenges reported by teams. In the upcoming cohort, the program leaders are planning to meet individually with each team and their manager prior to the workshops to strengthen the management's support and discuss alignment with the unit's priorities and time commitments.

## 7 CONCLUSION

The KT Challenge program has supported nurses to lead evidence‐based practice changes within their healthcare organisations, thus improving knowledge mobilisation to enhance patient care. The KT Challenge has continued to garner interest from other health care organisations looking for promising ways to empower nurses to be leaders in their fields and enhance their workforce's capacity for evidence‐informed care.

## FUNDING INFORMATION

The Knowledge Translation Challenge program is supported by Providence Health Care Professional Practice Office, St Paul's Foundation, Vancouver Coastal Health Research Institute, Robert H. N. Ho Enhancing Patient Care Fund supported by the VGH & UBC Hospital Foundation, and BC Cancer.

## CONFLICT OF INTEREST STATEMENT

The authors declare that they have no conflict of interests.

## PEER REVIEW

The peer review history for this article is available at https://www.webofscience.com/api/gateway/wos/peer‐review/10.1111/jan.16362.

## Data Availability

Data available on request due to privacy/ethical restrictions.

## References

[jan16362-bib-0001] Black, A. T. , Balneaves, L. G. , Garossino, C. , Puyat, J. H. , & Qian, H. (2015). Promoting evidence‐based practice through a research training program for point‐of‐care clinicians. The Journal of Nursing Administration, 45(1), 14–20. 10.1097/NNA.0000000000000151 25390076 PMC4263611

[jan16362-bib-0002] Black, A. T. , Steinberg, M. , Chisholm, A. E. , Coldwell, K. , Hoens, A. K. , Koh, J. C. , LeBlanc, A. , Mackay, M. , Salmon, A. , & Snow, M. E. (2021). Building capacity for implementation–The KT challenge. Implementation Science Communications, 2(1), 84. 34321107 10.1186/s43058-021-00186-x PMC8316705

[jan16362-bib-0003] Bolton, P. (2011). Improving physical health monitoring in secondary care for patients on clozapine. The Psychiatrist, 35(2), 49–55. 10.1192/pb.bp.109.028753

[jan16362-bib-0004] Bauer, M. S. , Damschroder, L. , Hagedorn, H. , Smith, J. , & Kilbourne, A. M. (2015). An introduction to implementation science for the non‐specialist. BMC Psychology, 3(1), 32. 10.1186/s40359-015-0089-9 26376626 PMC4573926

[jan16362-bib-0005] Casey, M. , O' Leary, D. , & Coghlan, D. (2018). Unpacking action research and implementation science: Implications for nursing. Journal of Advanced Nursing, 74(5), 1051–1058. 10.1111/jan.13494 29098709

[jan16362-bib-0006] Damschroder, L. J. , Reardon, C. M. , Widerquist, M. A. O. , & Lowery, J. (2002). The updated consolidated framework for implementation research based on user feedback. Implementation Science, 17, 75. 10.1186/s13012-022-01245-0 PMC9617234 36309746

[jan16362-bib-0007] De Berardis, D. , Serroni, N. , Campanella, D. , Olivieri, L. , Ferri, F. , Carano, A. , Cavuto, M. , Martinotti, G. , Cicconetti, A. , Piersanti, M. , Saverio Moschetta, F. , & Di Giannantonio, M. (2012). Update on the adverse effects of clozapine: Focus on myocarditis. Current Drug Safety, 7(1), 55–62. 10.2174/157488612800492681 22663959

[jan16362-bib-0008] Durlak, J. , & Dupre, E. (2008). Implementation matters: A review of research on the influence of implementation on program outcomes and the factors affecting implementation. American Journal of Community Psychology, 41, 327–350. 10.1007/s10464-008-9165-0 18322790

[jan16362-bib-0500] Eccles, M. , & Mittman, B. S. (2006). Welcome to Implementation Science. Implementation Science, 1(1), 1–3.

[jan16362-bib-0009] Field, B. , Booth, A. , Ilott, I. , & Gerrish, K. (2014). Using the knowledge to action framework in practice: A citation analysis and systematic review. Implementation Science, 9, 172. 10.1186/s13012-014-0172-2 25417046 PMC4258036

[jan16362-bib-0010] Gessner, B. , Carter, M. , Rahnama, K. , Almeida, A. , Borralho, C. , Mihic, T. , Ng, J. C. Y. , Puyat, J. H. , Russolillo, A. , & Halpape, K. C. (2024). Clozapine clinical toolkit optimizes inpatient clozapine monitoring. Mental Health Clinician, 14(2), 85–91. 38694883 10.9740/mhc.2024.04.085 PMC11058321

[jan16362-bib-0011] Graham, I. D. , Logan, J. , Harrison, M. B. , Straus, S. E. , Tetroe, J. , Caswell, W. , & Robinson, N. (2006). Lost in knowledge translation: Time for a map? Journal of Continuing Education in the Health Professions, 26(1), 13–24. 16557505 10.1002/chp.47

[jan16362-bib-0012] IAP2 Spectrum of Public Participation . (2018). IAP2 International Federation. https://www.iap2.org/page/pillars

[jan16362-bib-0013] Leitch, R. , & Day, C. W. (2000). Action research and reflective practice: Towards a holistic view. Educational Action Research, 8(1), 179–193. 10.1080/09650790000200108

[jan16362-bib-0014] Lingard, L. , Albert, M. , & Levinson, W. (2008). Grounded theory, mixed methods, and action research. BMJ (Clinical Research Ed.), 337, a567. 10.1136/bmj.39602.690162.47 18687728

[jan16362-bib-0015] Michie, S. , van Stralen, M. M. , & West, R. (2011). The behaviour change wheel: A new method for characterising and designing behaviour change interventions. Implementation Science, 6, 42 Accessed at: https://www.ncbi.nlm.nih.gov/pmc/articles/PMC3096582/ 21513547 10.1186/1748-5908-6-42 PMC3096582

[jan16362-bib-0016] Mitchell, A. , Delaffon, V. , & Lord, O. (2012). Let's get physical: Improving the medical care of people with severe mental illness. Advances in Psychiatric Treatment, 18(3), 216–225. 10.1192/apt.bp.111.009068

[jan16362-bib-0017] Mlambo, M. , Silén, C. , & McGrath, C. (2021). Lifelong learning and nurses' continuing professional development, a metasynthesis of the literature. BMC Nursing, 20(1), 62. 10.1186/s12912-021-00579-2 33853599 PMC8045269

[jan16362-bib-0018] National Academies of Sciences, Engineering, and Medicine [NASEM] . (2021). The future of nursing 2020–2030: Charting a path to achieve health equity. The National Academies Press. 10.17226/25982 34524769

[jan16362-bib-0019] National Institute of Care Excellence . (2014). Schizophrenia. Core interventions in the treatment and management of schizophrenia. National Institute for Clinical Excellence.

[jan16362-bib-0020] Orrell, K. , Yankanah, R. , Heon, E. , & Wright, J. G. (2015). A small grant funding program to promote innovation at an academic research hospital. Canadian Journal of Surgery, 58(5), 294–295. 10.1503/cjs.001915 PMC4599988 26384144

[jan16362-bib-0021] Pringsheim, T. , & Addington, D. (2017). Canadian schizophrenia guidelines: Introduction and guideline development process. Canadian Journal of Psychiatry, 62(9), 586–593. 10.1177/0706743717719897 28789558 PMC5593245

[jan16362-bib-0022] Rana, R. , Caron, M. P. , & Kanters, S. (2022). Nurse mentored, student research in undergraduate nursing education to support evidence‐based practice: A pilot study. Nursing Forum, 57(2), 225–233. 10.1111/nuf.12667 34713907

[jan16362-bib-0700] Roberts, N. A. , Young, A. M. , & Duff, J. (2023). Using Implementation Science in Nursing Research. Seminars in Oncology Nursing, 39. 10.1016/j.soncn.2023.151399 36894448

[jan16362-bib-0023] Ronaldson, K. J. , Fitzgerald, P. B. , & McNeil, J. J. (2015). Clozapine‐induced myocarditis, a widely overlooked adverse reaction. Acta Psychiatrica Scandinavica, 132(4), 231–240. 10.1111/acps.12416 25865238

[jan16362-bib-0024] Ruco, A. , Nichol, K. , Morassaei, S. , Bola, R. , & Di Prospero, L. (2021). Supporting discovery and inquiry: A Canadian hospital's approach to building research and innovation capacity in point‐of‐care health professionals. Quality Management in Health Care, 30(4), 267–275. 10.1097/QMH.0000000000000294 33843828

[jan16362-bib-0025] Russell‐Babin, K. , Friesen, M. A. , O'Brien, A. M. , McLaughlin, M. K. , Messing, J. , Mowery, B. , Bettencourt, A. P. , & Graling, P. R. (2023). A nurse‐led implementation science specialist program. The American Journal of Nursing, 123(12), 38–45. 10.1097/01.NAJ.0000997228.84722.c7 37988023

[jan16362-bib-0026] Santos, W. J. , Graham, I. D. , Lalonde, M. , Demery Varin, M. , & Squires, J. E. (2022). The effectiveness of champions in implementing innovations in health care: A systematic review. Implementation Science Communications, 3(1), 80. 10.1186/s43058-022-00315-0 35869516 PMC9308185

[jan16362-bib-0027] Westerlund, A. , Sundberg, L. , & Nilsen, P. (2019). Implementation of implementation science knowledge: The research‐practice gap paradox. Worldviews on Evidence‐Based Nursing, 16(5), 332–334. 10.1111/wvn.12403 31603625 PMC6899530

[jan16362-bib-0028] White, C. L. (2011). Nurse champions: A key role in bridging the gap between research and practice. Journal of Emergency Nursing, 37(4), 386–387. 10.1016/j.jen.2011.04.009 21621832

[jan16362-bib-0600] Zullig, L. L. , Deschodt, M. , & DeGeest, S. (2020). Embracing Implementation Science: A paradigm shift for nursing research. Journal of Nursing Scholarship, 52(1), 1–5. 10.1111/jnu.12507 31811696

